# Possibilities of Overcoming Resistance to Osimertinib in NSCLC Patients with Mutations in the *EGFR* Gene

**DOI:** 10.3390/cancers17040563

**Published:** 2025-02-07

**Authors:** Marcin Nicoś, Anna Sroka-Bartnicka, Ewa Kalinka, Paweł Krawczyk

**Affiliations:** 1 Department of Pneumonology, Oncology and Allergology, Medical University of Lublin, 20-059 Lublin, Poland; krapa@poczta.onet.pl; 2 Independent Unit of Spectroscopy and Chemical Imaging, Medical University of Lublin, 20-059 Lublin, Poland; anna.sroka-bartnicka@umlub.pl; 3 Department of Oncology, Polish Mother’s Memorial Hospital-Research Institute, 93-338 Lodz, Poland; ewakalinka@wp.pl

**Keywords:** osimertinib, EGFR TKIs, NSCLC, resistance

## Abstract

The advancement of genetic research has changed the treatment management of non-small cell lung cancer (NSCLC) and opened the era of personalized medicine. Currently, three generations of EGFR tyrosine kinase inhibitors (TKIs) are used in the treatment of NSCLC patients with activating mutations in the *EGFR* gene, and ongoing clinical trials examine the safety and effectiveness of new third and fourth generations. Osimertinib, a third generation of TKIs that binds irreversibly to abnormal tyrosine kinase, may be applied in various indications in patients with NSCLC: (i) in the second and subsequent lines of therapy in patients with resistance to first-generation or second-generation EGFR TKIs, (ii) in the first line of treatment in monotherapy in NSCLC patients with frequent or rare *EGFR* mutations, (iii) in combination with chemotherapy in patients with locally advanced or metastatic NSCLC with frequent *EGFR* mutations, (iv) in consolidation therapy in patients with locally advanced NSCLC who had previously received chemoradiotherapy, (v) in adjuvant treatment of NSCLC patients with stage IB—IIIA undergoing radical surgical resection. Despite the high efficacy of osimertinib in NSCLC patients harboring *EGFR* mutations, resistance driven in EGFR-dependent or EGFR-independent mechanisms may occur. Since resistance to osimertinib is poorly understood, the following review presents the overview of resistance mechanisms to osimertinib, methodological approaches for the resistance diagnosis, and the up-to-date treatment possibilities for overcoming the resistance process.

## Simple Summary

Currently, three generations of EGFR tyrosine kinase inhibitors (TKIs) are used in the treatment of non-small-cell lung cancer (NSCLC) patients with activating mutations in the *EGFR* gene, and ongoing clinical trials examine the safety and effectiveness of new third- and fourth-generations. Osimertinib, a third generation of TKIs that binds irreversibly to abnormal tyrosine kinase, may be applied in various indications in patients with NSCLC. Despite the high efficacy of osimertinib in NSCLC patients harboring *EGFR* mutations, resistance driven in EGFR-dependent or EGFR-independent mechanisms may occur. Since resistance to osimertinib is poorly understood, the following review presents the overview of resistance mechanisms to osimertinib, methodological approaches for the resistance diagnosis, and the up-to-date treatment possibilities for overcoming the resistance process.

## 1. Introduction

Until recently, non-small cell lung cancer (NSCLC) was nucleoplasm associated with an inferior prognosis that has changed with the advancement of genetic research, such as the discovery of driver mutations and the development of molecularly targeted therapies [1]. The first target for molecularly targeted therapies in NSCLC patients was the abnormal *EGFR* protein (epidermal growth factor receptor) arising from activating mutations in exons 18–21 of the *EGFR* gene [2]. As an effect, excessive and autonomous activation of EGFR tyrosine kinase occurs, which leads to the activation of signaling pathways and excessive proliferation of cancer cells. In turn, the effective inhibition of abnormal EGFR tyrosine kinase activity leads to apoptosis of these cells, which were affected by activating [2,3]. Activating mutations in the *EGFR* gene occur in about 10% of Caucasian patients and 40% of Asian patients and are more frequently observed in women and young, nonsmoking patients with lung adenocarcinoma (LUAD) [4]. Three generations of EGFR tyrosine kinase inhibitors (TKIs) have been used in the treatment of NSCLC patients with activating mutations in the *EGFR* gene—the first generation is represented by reversible erlotinib and gefitinib [5], the second generation by irreversible afatinib [6], and the third generation by irreversible osimertinib and lazertinib [7]. Clinical trials are ongoing to examine the safety and effectiveness of new third- and fourth-generation EGFR TKIs [8].

Osimertinib, which preferentially binds irreversibly to abnormal tyrosine kinase, particularly in patients with more common mutations such as L858R substitution in exon 21 or an exon 19 deletion, has been used in the treatment of NSCLC patients in 2015. Currently, it can be used in five indications in patients with NSCLC [9]. Osimertinib treatment may be applied in the second and subsequent lines of therapy in patients with resistance to first-generation or second-generation EGFR TKIs who acquire a secondary T790M mutation in exon 20 of the *EGFR* gene that was registered based on the results of the phase III AURA3 study, comparing the efficacy of osimertinib to chemotherapy [10]. On the other hand the FLAURA study compared the efficacy of osimertinib monotherapy with the efficacy of standard of care (SoC), i.e., erlotinib or gefitinib therapy in patients with advanced NSCLC with frequent *EGFR* mutations (deletions in exon 19 or L858R substitution in exon 21) indicating the applicability of osimertinib in the first line of treatment in monotherapy [11]. Moreover, osimertinib monotherapy may also be used in patients with rare mutations in exons 18–21 of the *EGFR* gene (except patients with insertions in exon 20) and in patients with locally advanced NSCLC without the possibility of radical treatment [12]. The FLAURA2 study recently showed the efficacy of osimertinib in combination with chemotherapy compared to osimertinib monotherapy in patients with locally advanced or metastatic NSCLC with frequent *EGFR* mutations. Moreover, the results indicated a reduction in the risk of progression, favouring combination therapy [13]. Further, the LAURA study tested osimertinib in consolidation therapy in patients with locally advanced NSCLC who had previously received chemoradiotherapy, indicating a significant reduction of the risk of progression in patients receiving osimertinib rather than placebo [14]. Lastly, osimertinib has also been used in adjuvant treatment of patients with stage IB—IIIA NSCLC undergoing radical surgical resection (lobectomy or pneumonectomy). The ADAURA study included patients with common *EGFR* mutations who could receive adjuvant chemotherapy before osimertinib therapy, showing that osimertinib, compared to placebo, brought a highly significant reduction in the risk of disease recurrence in all studied subgroups [15].

Despite the high efficacy of osimertinib, which translates into a reduced risk of disease progression or relapse, resistance to osimertinib may occur in NSCLC patients with activating mutations in the *EGFR* gene [16,17]. The causes of resistance to osimertinib are more complex and poorly understood than the causes of resistance to earlier generations of EGFR TKIs, who usually acquire the T790M mutation [18,19,20]. In addition, the type of resistance depends on the line of treatment and the duration of osimertinib therapy [21]. Considering those aspects, in this review, we present the overview of resistance mechanisms to osimertinib and methodological approaches for diagnosing the resistance. Ultimately, we highlight the up-to-date treatment possibilities for overcoming this resistance process, indicating the future perspectives.

## 2. Mechanism of Resistance to Osimertinib

**Figure 1:**
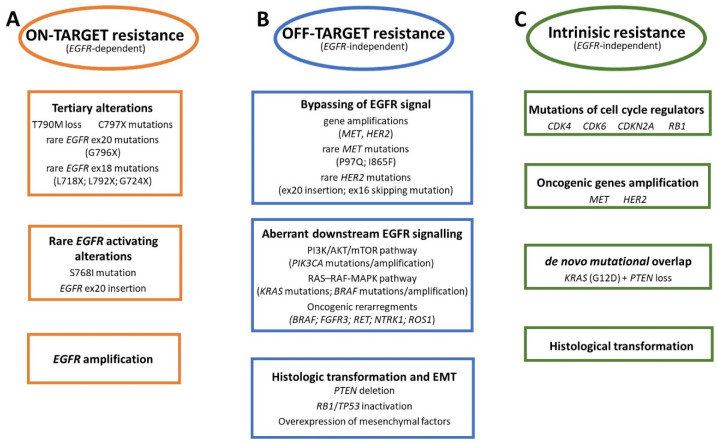
The overview of acquired and intrinsic resistance mechanism to osimertinib. (**A**)—illustrate the *EGFR*-dependent, on-target resistance mechanisms induced by the tertiary *EGFR* alterations, rare activating *EGFR* mutations and *EGFR* amplification. (**B**)—presents the *EGFR*-independent, off-target resistance mechanisms stimulated by alterations activating cellular signals bypassing the EGFR cascade, affecting downstream EGFR signalling, histologic transformation, and epithelial-to-mesenchymal transition (EMT). (**C**)—shows intrinsic and *EGFR*-independent resistance mechanisms driven by mutations in genes regulating the cell cycle, oncogenic amplifications, de novo overlap of oncogenic alterations, and histological transformation of lung adenocarcinoma (LUAD) to small-cell lung cancer (SCLC).

Despite the success of osimertinib in the first- and second-line of treatment, both the acquired and intrinsic resistance limits a prolonged clinical benefit [17]. The acquired resistance may be driven by *EGFR*-linked (on-target, Figure 1A) or *EGFR*-independent mechanisms (off-target, Figure 1B) involving activation of alternative bypass pathways, aberrant downstream EGFR signalling or histologic transformation [16,19]. Interestingly, the on-target resistance mechanisms appear both in the first- and second-line settings, while the off-target mechanism may be more relevant in first-line therapy than in further lines of treatment [16,20]. However, the on-target and off-target aberrations can co-occur within the same tumor and co-exist with *EGFR* tertiary mutations that arise from the complexity and heterogeneity of cancer evolution driven by EGFR TKI treatment [17,20]. Hence, repeated tumor biopsies or plasma genotyping at the time of progression on osimertinib are crucial steps in unravelling resistance mechanisms and guiding future therapies [16]. On the other hand, the intrinsic mechanism is also driven by off-target processes (Figure 1C). It concerns some subset of NSCLC and may be associated with alterations in cell-cycle regulators such as CDK4, CDK6, CDKN2A or RB1 that affect even 12% and 10% of patients upon progression to osimertinib administered as a second-line and first-line therapy, respectively [10,18,22].

### 2.1. On-Target Resistance Mechanisms

**Figure 2:**
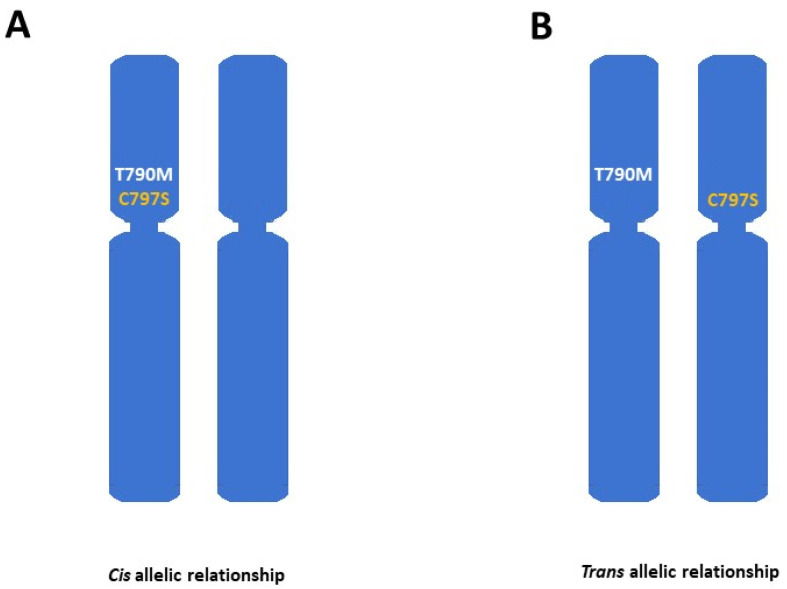
The overview of the allelic coexistence between T790M and C797S substation that impacts the anti-EGFR response. (**A**)—illustrates the overlap between T790M and C797S mutations at the same allele (in cis position) that cause resistance to all available EGFR TKIs. (**B**)—presents the co-existence of T790M and C797S at different alleles (in *trans* position) that determines the resistance of the tumor to osimertinib.

Primarily, osimertinib was administrated as the sequential treatment regimen if the substitution T790M in exon 20 of the *EGFR* gene was found at the moment of progression to the first (gefitinib, erlotinib) or second (afatinib, dacomitinib) generation of TKIs [23]. At that moment, it was thought that the osimertinib efficiency was determined by T790M substitution that increases the osimertinb binding with the cysteine-797 (C797) in the ATP pocket of the EGFR protein [17,24]. However, osimertinib efficiently binds with the C797 regardless of the presence of T790M mutation and thus is administrated in the first line of untreated *EGFR*-positive NSCLC patients [22]. Following this knowledge, both T790M- and C797-dependent alterations are the most relevant drivers of on-target resistance to osimertinib. Oxnard et al. suggested that early or late resistance to the second line of osimertinib may be lined with T790M lost or retention, respectively [25]. The AURA3 trial [10] indicated that at the moment of progression to osimertinib in the second-line therapy, even 49% of NSCLC patients lost the T790M substitution and 21% acquired the tertiary *EGFR* mutations such as C797S or C797R. Interestingly, after progression, even 83% of patients who lost T790M maintained activating *EGFR* mutations, while all patients who acquired tertiary C797X *EGFR* mutations retained the T790M substitution [10]. In such a scenario, the treatment failure may be associated with alternative competing off-target resistance [16,17]. On the other hand, the FLAURA trial on the NSCLC cohort receiving the frontline osimertinib treatment, C797S (rarely C797G) represents a key mechanism of resistance, regardless of pre- or post-progression T790M appearance [22]. C797X mutations enable resistance to osimertinib in any line of therapy [17,24,26]. However, the allelic context of C797S may predict the responsiveness to alternative treatments. In particular, C797S and T790M mutations more commonly appear in the cis position (at the same allele, Figure 2A), leading to resistance to all available EGFR TKIs [17,26,27]. However, if the C797S and T790M mutations are in the *trans* position (at different alleles, Figure 2B), the tumor cells resist osimertinib. Still, they may be sensitive to a combination of first- and third-generation TKIs. Genotyping of *EGFR* mutations in plasma samples at the moment of progression could predict the type of resistance mechanisms [25].

Also, some other tertiary rare *EGFR* mutations, mainly localised adjacent to C797 position G796 at exon 20th (G796R, G796S and G796D), or in exon 18 (L718, L792, G724) [28,29,30], have been associated with the resistance process to osimertinib, regardless of their coexistence with T790M [21,22,31] and C797S substations [28,32]. However, in some cases, G724S mutation selectively confers resistance and sensitivity to osimertinib when overlapping with exon 19 deletion and L858R substitution, respectively [17,33]. In addition to *EGFR* tertiary mutations, other rare activating mutations such as S768I or insertion in exon 20 of the *EGFR* gene were indicated in patients after progression to osimertinib both in the first and second lines of treatment [10,22,33]. *EGFR* amplification, which was reported in 19–29% of osimertinib-resistant NSCLC patients [21,34], may drive the resistance process by aberrant activation of several downstream signalling pathways such as RAS/RAF/MAPK, PI3K/AKT/mTOR and STAT [34]. Data suggest that *EGFR* amplification may drive resistance in NSCLC patients who retained T790M substitution [10,21,25] and that *EGFR* amplification is more common in acquired resistance to osimertinib [35,36]. However, *EGFR* amplification often occurs concurrently with activating *EGFR* mutations, thus, it cannot determine whether *EGFR* amplification is a primary or acquired mechanism of resistance to osimertinib [37].

### 2.2. Off-Target Resistance Mechanisms

Bypassing EGFR inhibition is one of the most common *EGFR*-independent acquired resistance mechanisms. Commonly, it is driven by *MET* amplification, which was also reported as a potential mechanism of intrinsic resistance to osimertinib [10,38,39]. *MET* amplification in the AURA3 study was observed in nearly 19% of the samples resistant to the second-line osimertinib (in 7% of cases, *MET* amplification co-occurred with C797S mutation) and was also lined with *CDK6* and *BRAF* amplifications [10,21]. Moreover, *MET* amplification was the most common resistance mechanism encountered in 15% of patients receiving frontline-based therapy [22]. There are some suggestions that rare *MET* gene hot-spot mutations (P97Q, I865F substitutions) may trigger resistance to the second line of osimertinib therapy [28]. In contrast, the impact of skipping *MET* exon 14 (METex14) mutation on resistance processes remains unknown [40]. Other off-target resistance mechanisms bypassing EGFR inhibition may be driven through HER proteins that belong to the EGFR family [41]. Amplification of *HER2* was indicated as mutually exclusive with the T790M mutation [10,28,41], affecting 2% of cases of first-line osimertinib [22] and 5% of patients who developed resistance to second-line osimertinib [10], respectively. Rare insertions in exon 20 and exon 16-skipping mutation in the *HER2* gene were also implicated in resistance to osimertinib [42]. Moreover, there are some premises that *HER2* amplification and HER3 overexpression may drive intrinsic resistance to osimertinib [16,18].

Further off-target resistance may be driven by aberrant downstream EGFR signalling affecting various common cancer-related pathways [16,17,18,43]. PI3K/AKT/mTOR dysregulation was observed in 4–11% of patients who progressed to osimertinib in the frontline and second-line of treatment [10,21,25,39,44,45]. It was driven mainly by common *PIK3CA* gene mutations (E454K, E542K, R88Q, N345K, and E418K) [28,45,46]. Moreover, *PIK3CA* amplifications co-occurred with *HER2* amplifications within the AURA3 study [10]. On the other hand, *PTEN* deletion, which also affects the PI3K/AKT/mTOR pathway, was commonly associated with histologic transformation of osimertinib-resistant LUADs to LUSC or SCLC [28,47]. Aberrant downstream signalling in the RAS/RAF/MAPK pathway also leads to osimertinib resistance in NSCLC patients [38]. It may be driven by *BRAF* and *KRAS* gene mutations detected by FLAURA and AURA3 trials in patients resistant to the frontline and second-line osimertinib therapy [10,22]. The *KRAS* G12S mutation mainly acquires resistance to second-line osimertinib [21,25]. At the same time, *KRAS* G12D triggers osimertinib failure both in the first-line and in subsequent lines of therapy [10,22,43]. Furthermore, there is evidence that pre-existing de novo *KRAS* G12D mutations associated with *PTEN* loss were observed in patients with intrinsic resistance to osimertinib in the second-line of treatment [43]. Finally, *BRAF* gene alterations (substitutionV600E, gene amplifications and *AGK–BRAF* or *ESYT2–BRAF* fusions) were detected upon progression to the first- or second-line of osimertinib [22,48,49]. There are also proofs that chromosomal rearrangements within driver oncogenes, such as *FGFR3*, *RET*, *NTRK1*, and *ROS1* have been identified in 3–10% of cases of acquired resistance to second-line osimertinib [10,21,25,50]. In particular, the AURA 3 study indicated that *FGFR3–TACC3* and *RET–ERCC1* co-occurred with *EGFR* C797S mutation, *BRAF* mutation, and *MET* amplification. [10]. There is also evidence that the fusion *SPTBN1–ALK* has been detected in a single patient who received frontline osimertinib but not in patients treated with second-line [10,22].

The histologic transformation from NSCLC to squamous cell carcinoma or small-cell lung cancer (SCLC) is always associated with a poor prognosis [51], even if initial *EGFR*-sensitising mutations are retained by transformed subtypes [48,52]. This phenomenon may be driven by complete inactivation of the tumour-suppressor genes such as *RB1* and *TP53* [53,54], and was identified as an essential reason for resistance to osimertinib that affects 15% and 14% of patients receiving osimertinib in frontline or later lines, respectively [53,55]. Moreover, in some reports, SCLC transformation is a putative mechanism of intrinsic resistance to osimertinib [56]. Epithelial-to-mesenchymal transition (EMT) is another phenomenon observed in NSCLC with acquired resistance to osimertinib [57,58]. The EMT process may implicate specific pathways that may trigger drug-related resistance [57]. However, the EMT comes with increased cell motility by losing adherens junctions and overexpression of the mesenchymal biomarkers, a hallmark of distant cancer dissemination and indicates that the cancer process has escaped treatment control [53,54].

## 3. Diagnosis of Osimertinib Resistance

The resistance mechanisms to osimertinib therapy are highly complex. Thus, diagnosing individual genetic abnormalities using single-gene tests, such as real-time PCR (polymerase chain reaction), FISH (fluorescence in situ hybridization), or immunohistochemical methods, is complex and ineffective [16,59]. On the other hand, single-gene tests are cheap, quick to perform, do not require a large number of cancer cells, and are not sensitive to nucleic acid damage, while the next-generation sequencing (NGS) technique enables simultaneous profiling of multiple genes, therefore, it is the most commonly used to diagnose resistance causes [59,60]. Comprehensive genomic profiling (CGP) is more often performed for this purpose than whole exome sequencing (WES). Currently, there is no evidence to suggest that whole genome sequencing (WGS) should be used to diagnose resistance mechanisms to osimertinib [60]. Liquid biopsy (peripheral blood) is the most often tested material used in NGS-based diagnostics. However, the percentage of patients with valid NGS results from circulating tumor DNA (ctDNA) is unsatisfactory since ctDNA is usually a tiny fraction of circulating free DNA (cfDNA) [59,61,62]. The amount of isolated ctDNA from 5 mL of peripheral blood usually does not exceed 30 ng. In addition, ctDNA has a short half-life (from several minutes to several hours) and is reliably shorter than normal cfDNA [63]. Furthermore, studying genetic rearrangements in some NGS platforms requires using RNA, which is highly unstable in liquid biopsy [59,60]. Therefore, in some patients, ctDNA is not sufficient material to perform an NGS test [60,61].

Biopsy material from primary tumor or metastases provides more neoplastic DNA than liquid biopsy [64]. However, the genetic changes detected in a single biopsy do not represent the entire neoplastic process, and not considering its heterogeneity that is avoided by ctDNA testing [64,65]. Moreover, material collected from neoplastic lesions may contain too few cancer cells (especially in material form ultrasound-guided fine-needle biopsy), and nucleic acids may be damaged during the fixation process (formalin-fixed and paraffin-embedded, FFPE) [61,66]. Although performing single-gene tests is not recommended for the comprehensive diagnosis of osimertinib resistance, it may be necessary to perform in selected patients, especially when qualification for clinical trials based on their results [59]. Single-gene tests, except selected real-time PCR tests, are mainly performed using FFPE materials. Among the on-target resistance mechanisms, the presence of C797S, C797N, G796R, G724S, L871Q, L718Q, and L718V mutations can be tested using the real-time PCR technique [28,29,30]. In contrast, the detection of T790M, including T790M loss and C797S mutations, can be performed by various NGS and PCR-based commercially available tests [60,67,68,69]. In contrast, *EGFR* gene amplification, another on-target resistance mechanism, may be detected using the FISH technique [70]. Moreover, the FISH technique can be used to examine the amplification of the *MET*, *HER2*, *PIK3CA*, *CDKN2A*, and cyclin-encoded genes [62,71] that drives the off-target resistance mechanisms to osimertinib. Further, the real-time PCR technique can examine other off-target alterations in the *KRAS*, *NRAS*, *BRAF*, *HER2*, and *PIK3CA* genes that harbor the most common oncogenic mutations [62]. However, FFPE materials would not be sufficient to analyse all the above-mentioned alterations. Therefore, all of these genetic alterations should be simultaneously examined using the NGS technique in tissue or liquid biopsy, which additionally allows for the examination of genetic rearrangements involving the *ALK*, *RET*, *BRAF*, *NTRK*, *RET*, *ROS1*, *FGFR3*, and *MET* genes [38,44,60]. Moreover, many rare genetic alterations, such as insertions in exon 20 of the *HER2* gene, mutations in other than 600 codons of the *BRAF* gene, rare substitutions in exon 20 of the *EGFR* gene, and rare rearrangements of the *ALK*, *ROS1*, *NTRK*, *BRAF* genes, and others can only be detected by NGS [16,17,44,60].

*MET* gene amplification can occur as a focal amplification of the gene number or result from chromosome 7 polysomy and was initially studied using the FISH technique [72,73,74]. Focal MET gene amplification is the most common driver alteration for cancer [72,75], and it is recognized as the most common cause of osimertinib resistance [16,17,74]. This assay was used in the TATTON clinical trial, which examined the possibility of overcoming osimertinib resistance by adding savolitinib (MET inhibitor) to osimertinib [76]. In the FISH method, *MET* amplification is defined as the presence of *MET* gene copy number of five or as the ratio of the *MET* gene copy number to the number of chromosome 7 centromeres (CEP7) of two or more. Despite its advantage in simultaneously identifying multiple genetic abnormalities, NGS cannot assess the number of CEP7 [74,77]. Thus, the increase in the *MET* gene copy number pattern in the NGS method may result from both *MET* gene amplification and chromosome 7 polysomy [74]. Therefore, the FISH test is still recommended for the assessment of *MET* gene amplification as well as *HER2* gene amplification due to similar remarks [17,74,76,78]. Immunohistochemical tests diagnose the causes of resistance associated with SCLC transformation (expression of CD56, Ki-67, neuroendocrine markers on cancer cells) [55,56] and epithelial to mesenchymal transition (EMT) [57]. EMT-associated alterations affect the decrease in E-cadherin and the overexpression of vimentin (mesenchymal biomarker) or Hakai protein [57,58]. Furthermore, the overexpression of MET or HER2 proteins and ALK abnormal protein can also be determined by IHC methods [78].

Diagnosing acquired resistance mechanisms to osimertinib therapy was performed for the first time in NSCLC patients with mutations in the *EGFR* gene and with resistance to first- or second-generation EGFR TKIs who received osimertinib in the further lane of treatment [16,17,19]. In the phase III AURA3 study, patients with acquired T790M mutation in exon 20 of the *EGFR* gene and in progression after therapy with erlotinib, gefitinib or afatinib received osimertinib or chemotherapy in the second-line of treatment [10]. Among 279 patients treated with osimertinib, only 78 (27.9%) were evaluable for resistance mechanism analysis using the NGS technique, which means that patients had detectable *EGFR* mutations in plasma at baseline and had paired plasma samples from baseline and at progression or treatment discontinuation [79,80]. ctDNA extracted from paired plasma samples was analyzed using a 74-gene NGS panel (Guardant Health, Guardant 360 assay) [81]. The variant allelic fraction (VAF) limit detected was 0.04–0.06%, and 32 of 78 patients (41%) had at least one detectable genetic abnormality that could be responsible for resistance to osimertinib. Acquired *EGFR* mutations (mainly C797X) or MET gene amplification were detected in 17 and 14 patients (22% and 18%), respectively [10,79]. *HER2* gene amplification and rearrangements of *FGFR3*, *NTRK1*, and *RET* genes were detected in single patients, while *PIK3CA* gene amplification was detected in three patients (4%). In 39 patients (50%), the T790M mutation was not detected in plasma samples, which could be responsible for losing sensitivity to osimertinib [10,69,80]. Moreover, acquired mutations in the *EGFR* gene co-occurred with *MET* gene amplification in five patients, and in two patients, the V600E mutation in the *BRAF* gene and the G12D mutation in the *KRAS* gene were also detected [10]. *MET* and *HER2* amplifications were associated with cell cycle gene alterations in some patients. Moreover, *HER2* amplification was also associated with *PIK3CA* amplification [80].

In phase III FLAURA study [22], NGS approaches based on Guardant Health, Guardant 360 74 genes panel or Guardant OMNI 500 genes panel [81] were applied to 137 of 279 patients (48%) patients treated with first-line osimertinib therapy who had paired plasma samples at treatment baseline and disease progression or treatment discontinuation [22,79]. With the VAF 0.04–0.06%, 38 of 109 patients (39%) had a detectable acquired genetic abnormality that may be responsible for resistance to osimertinib. More than one acquired genetic alteration was detected in 15 patients (14%). *MET* amplification was the most common (16% of patients) acquired resistance alteration following mutations in the *EGFR* gene (10% of patients, including C797S mutation in 6%). Mutations in the *PIK3CA* gene, amplifications of genes regulating the cell cycle (*CDK6*, *CDK4*, *CCND1*), *BRAF* V600E mutation, *KRAS* gene mutations, *ALK* gene rearrangement, and *HER2* gene amplification were detected in individual patients [22,80], and the occurrence of these genetic abnormalities was not clearly associated with the duration of osimertinib treatment [79].

The data presented in the AURA3 and FLAURA clinical trials describe many difficulties in diagnosing genetic abnormalities responsible for osimertinib resistance [10,22]. Firstly, the profile of genetic alterations differs in patients treated with osimertinib in the first- and subsequent lines. Their appearance probably depends on the duration of osimertinib therapy [82]. Secondly, it is necessary to compare the results of genetic tests performed before treatment with those obtained at the time of progression or discontinuation of therapy. It should be considered that tumor tissue during progression is often unavailable [63,83], and liquid biopsy may not have been collected before treatment. Thirdly, the NGS technique best defines resistance mechanisms to osimertinib. However, it has some limitations, such as sensitivity that does not always allow for the detection of ctDNA in liquid biopsy (the percentage of patients whose ctDNA was tested using the NGS technique in the AURA3 and FLAURA clinical trials did not exceed 50% [10,22]. Furthermore, the NGS method is susceptible to DNA fragmentation (e.g., in FFPE materials), which is responsible for low coverage of the tested DNA and low depth of sequencing. In some cases, NGS is less valuable than FISH or IHC methods, such as in examining *MET* gene amplification, transformation to SCLC or the EMT phenomenon [38,43,44,60]. In summary, difficulties in diagnosis of the resistance mechanisms to osimertinib and their great diversity have caused genetic tests not to be routinely performed in qualification for subsequent lines of treatment after osimertinib therapy, especially since new molecularly targeted drugs are only available in clinical trials [16,17,79,80,82].

## 4. Amivantamab with Chemotherapy as an Approved Treatment Option for Osimertinib-Resistant Patients with *EGFR* Mutations

Based on the results of the MARIPOSA-2 study, amivantamab (bispecific antibody against EGFR and MET receptors) in combination with chemotherapy, is currently the only treatment manner with European Medicine Agency (EMA) and Food and Drugs Administration (FDA) approval (September 2024) for NSCLC patients after disease progression on osimertinib treatment [84]. The MARIPOSA-2 study recruited patients with locally advanced or metastatic NSCLC with a documented deletion in exon 19 or L858R substitutions in the *EGFR* gene. The study groups were well balanced—131 patients received a combination of amivantamab and chemotherapy, 263 amivantamab with lazertinib and chemotherapy, and 263 patients received chemotherapy alone [84,85]. Exploratory efficacy endpoints of the study were overall survival (OS), time to symptomatic progression (TTSP), time to treatment discontinuation (TTTD), time to subsequent therapy (TTST), progression-free survival (PFS), and PFS measured from the moment of starting the following line of treatment (PFS2). The median age of patients across the studied groups was 61–62 years, and Asian and Caucasian patients constituted approximately 48% of the study groups [85]. Approximately 60% of patients remained in a good performance status (PS 1) according to ECOG [86], more than 30% of patients had reported a history of smoking, and more than 40% of patients had a history of brain metastases (approximately half of them was receiving CNS radiotherapy). Approximately 70% of patients in the studied groups received osimertinib in the frontline therapy, of which 63–70% harbored deletion in exon 19 of the *EGFR* gene [84]. During the study, increased hematologic toxicity was observed in the group receiving amivantamab, lazertinib, and chemotherapy, which influenced the modification of the treatment regimen that lazertinib was administered after the completion of carboplatin therapy. An extension cohort has been initiated, including new patients, to assess the safety and efficacy of the modified regimen [85].

The median PFS evaluated by blinded independent central review (BICR) was 6.3 months in patients treated with amivantamab and chemotherapy, 8.3 months in patients who received amivantamab, lazertinib and chemotherapy, and 4.2 months in a cohort of patients treated with chemotherapy only. The risk of progression was significantly lower in the amivantamab and chemotherapy arm compared to the chemotherapy arm (HR = 0.48, 95% CI: 0.36–0.64, *p* < 0.001) and in the amivantamab, lazertinib and chemotherapy arm compared to the chemotherapy arm (HR = 0.44, 95% CI: 0.35–0.56, *p* < 0.001) [84,85]. In comparison, the median investigator-assessed PFS was 8.2 months, 8.3 months, and 4.2 months, respectively, with a reduction of progression risk of 59% and 62%, and 22%, 37%, and 13% of patients remained progression-free after one year of follow-up in the individual treatment groups. The median progression-free survival in the central nervous system (CNS) was 12.5 months in the amivantamab plus chemotherapy group, 12.8 months in the amivantamab, lazertinb and chemotherapy group, and 8.3 months in the chemotherapy alone group. The reduction in the risk of progression in CNS between the amivantamab groups versus the chemotherapy alone group was 45% and 42%. Moreover, 50%, 54%, and 34% of patients remained without progression in CNS after 1 year of follow-up in the individual treatment arms. The PFS benefit was consistent across groups with different clinical and demographic characterisations [84,85].

PFS measured from the moment of starting of the subsequent line of therapy (PFS2) was significantly prolonged in patients treated with amivantamab in combination with chemotherapy compared to patients who received chemotherapy alone (16 vs. 11.6 months, respectively, HR = 0.64, 95% CI: 0.48–0.85, *p* = 0.002). Moreover, 18-month survival without second progression was observed in 39% of patients in the first group and 27% in the second group [87,88]. Combined therapy insignificantly decreased the risk of death (HR = 0.77; 95% CI: 0.49–1.21) at the first interim OS analysis, as was presented at ESMO 2024 [87]. Second interim OS analysis at a median follow-up of 18.1 months showed that 50% of patients who received amivantamab and chemotherapy, while only 40% of patients receiving chemotherapy achieved 18-month survival (17.7 vs. 15.3 months; HR = 0.73; 95% CI: 0.54–0.99, *p* = 0.039) [84,85,87]. The summarize of the study outcomes is presented in the Table 1.

Further, in the MARIPOSA-2 study [84,85], adverse events (AEs) of grade 3 or higher such as neutropenia (including febrile neutropenia), thrombocytopenia, anaemia, and leukopenia were reported by 92% of patients who received amivantamab, lazertinib and chemotherapy, 72% of patients treated with amivantamab and chemotherapy, and 48% of patients undergoing only chemotherapy. Further, infusion-related reactions (IRRs) occurred in 58% and 56% of patients treated with amivantamab and chemotherapy or amivantamab, lazertinib, and chemotherapy. However, infusion-related severe toxicities occurred in 5% and 3% of such patients, respectively. Venous thromboembolism occurred in 10%, 22%, and 5% of patients (in grade ≥ 3 in 2%, 6% and 3%), respectively. Rash (including skin exfoliation) was observed in 71% and 75% of patients treated with amivantamab and chemotherapy, or amivantamab, lazertinib, and chemotherapy. Other skin toxicities of grade ≥3 occurred in 10% and 15% of patients, respectively. Severe interstitial lung diseases occurred in 6 (3%) patients receiving amivantamab. Dose interruptions, reductions, and discontinuations due to AEs (mainly hematologic toxicities) were required for 65%, 41%, and 18% of patients who received amivantamab and chemotherapy, 77%, 65%, and 34% of patients treated with amivantamab, lazertinib, and chemotherapy, and 33%, 15%, and 4% of patients undergoing only chemotherapy [84,85].

When amivantamab was administered intravenously, it was associated with an increased rate of infusion-related reactions. SKIPPirr, a phase 2 clinical trial [89], approaches to manage IRRs, including a split first dose of amivantamab over 2 days and premedication with antihistamines, antipyretics, and glucocorticoids. The study enrolled patients with advanced or metastatic NSCLC with *EGFR* exon 19 deletions or L858R mutation whose disease progressed on prior Osimertinib- and platinum-based chemotherapy. Prophylaxis with oral dexamethasone 8 mg for 2 days before infusion and another dose 1 h before infusion (5 total doses) led to a three-fold reduction in IRRs in patients treated with amivantamab. During the first administration of amivanatamb, IRRs occurred in 22.5% of patients receiving dexamethasone in a prophylactic dose of 8 mg (IRRs were grade 1–2). The most common IRR-related symptoms were nausea (8%), dyspnea (5%), and hypotension (5%) [89]. In comparison, IRRs in all study cohorts (n = 61) occurred in 46% of patients, including severe IRRs in 3%. Prophylaxis-related AEs in grades 1–2 occurred in 7% of patients receiving dexamethasone [90].

Minimising adverse events of amivantamab therapy was the goal of the phase III PALOMA-3 study [91]. It included patients with locally advanced or metastatic NSCLC that progressed on or after osimertinib- and platinum-based chemotherapy. Patients carrying a deletion in exon 21 or an L858R substitution in exon 21 of the *EGFR* gene received amivantamab subcutaneously (SC) or intravenously (IV) in combination with lazertinib.

Patients treated with SC amivantamab showed a five-fold reduction of IRRs (13% vs. 66%) compared to patients receiving IV amivantamab. The risk of venous thromboembolism was also reduced in patients receiving prophylactic anticoagulation compared to those without this prophylaxis, and substantially faster administration times occurred in patients receiving SC amivantamab (first administration < 5 min vs. 5.0 h; third administration < 5 min vs. 2.3 h). Third-line SC amivantamab demonstrated non-inferior efficacy compared to IV amivantamab. Median PFS in patients receiving SC amivantamab was 6.1 months, and in patients treated with IV amivantamab, 4.3 months (HR = 0.84, 95% CI: 0.64–1.1, *p* = 0.2), while median OS was not reached (HR = 0.62, 95% CI: 0.42–0.92, *p* = 0.02) [91].

## 5. Clinical Trials with New Drugs Overcoming the Resistance to Osimertinib (Efficacy and Safety)

As several mechanisms of resistance to osimertinib have been described, many new compounds targeting them specifically were designed. Other treatment approaches after progression on osimertinib also take advantage of the activity of antibody-drug conjugates (ADC) targeting biomarkers not linked with the resistance mechanism to osimertinib. Since multiple targeted therapies were tested in the progression setting after osimertinib, we followed the development of the most promising ones below.

### 5.1. Treatment Targeting the Mechanism of Resistance to Osimertinib Driven by MET Amplification and Overexpression or C797S Mutation

The TATTON phase I study was the first trial developing the combination of osimertinib and savolitinib, which started in 2015 [92]. The study enrolled 138 patients with progression after EGFR TKI treatment, and 69 of them were pretreated with a third-generation EGFR inhibitor, while 39 out of the 69 patients received at least three lines of therapy before study entry. MET dysregulation was an inclusion criterion diagnosed based on FISH or IHC methods. In these 69 patients, ORR with osimertinib combined with savolitinib was 33%, with a 75% disease control rate. It was translated into a median PFS of 5.5 months, a DoR of 9.5 months and a median OS of 30.3 months, with a 53% 18-month survival rate [76].

After the results of TATTON, the phase II, non-randomized, multicenter, open-label SAVANNAH trial [93] was initiated to test if the combination of 80 mg osimertinib and 300 mg of savolitinib is effective in patients progressing after osimertinib with *MET* alterations. As savolitinib is a selective MET tyrosine kinase inhibitor that targets *MET*-driven lung cancer, thus *MET* alterations were diagnosed at progression using a liquid or tissue biopsy. The study’s preliminary results indicated that 49% ORR was achieved in patients with high *MET* amplification (defined as ≥10 gene copies in the FISH method) and/or high overexpression (defined as overexpression of MET on 90% of cancer cells in the IHC method). In contrast, in those patients without high levels of *MET* alterations, the ORR reached 9% only. Moreover, chemotherapy-naïve patients had even 52% higher benefit of ORR. Median PFS in patients with high *MET* alterations was 7.1 months compared to 2.8 months in the subgroup with low *MET* alterations. The safety analysis showed no new signals for the studied compounds [77].

The preliminary encouraging results of the SAVANNAH trial led to the initiation of a phase III randomised SAFFRON trial comparing osimertinib combined with savolitinib to platinum plus pemetrexed chemotherapy in patients progressing after osimertinib [94]. The study currently enrols the cohort with *MET* amplification and/or MET overexpression in a post-progression tissue biopsy as one of the main inclusion criteria. The PFS is the primary endpoint of the study. At the same time, OS, ORR, quality of life, pharmacokinetics, disease control rate, time to discontinuation of treatment, tumor shrinkage, and duration of response are the secondary endpoints (NCT05261399) [95].

The next step in the development of the combination tested by SAVANNAH and SAFFRON trials is the initiation of a double-blind, multicenter phase III randomized SANOVO trial [96], which aims to assess the combination of osimertinib with savolitinib versus osimertinib in first-line treatment of *EGFR*-mutated advanced NSCLC patients with MET alterations with the hypothesis that the combination strategy would delay progression. The study was started in 2021 and is still ongoing. Its primary endpoint is PFS, while OS, ORR, DoR, safety, and tolerability are the secondary endpoints (NCT05009836) [95].

In 2023, Liam et al. published the final results of a randomized phase II INSIGHT trial [97] conducted in patients with advanced or metastatic NSCLC with *EGFR* mutations and with acquired *MET* dysregulation who progressed after the first-line treatment with the first or second generation of EGFR inhibitors. The biomarker inclusion criteria were: *MET* gene copy number (GCN) ≥ 5 or ratio of the *MET* gene copy number to the CEP7 number of ≥2:1 or MET overexpression with IHC 2+/3+. The 55 patients were randomised to tepotinib, a highly selective MET TKI, 500 mg daily combined with 250 mg of gefitinib daily versus chemotherapy. The median PFS was not significantly improved (4.9 months versus 4.4 months; stratified HR = 0.67, 90% CI: 0.35–1.28). However, in 19 patients with *MET* amplification, a clear benefit was confirmed for the tepotinib plus gefitinib combination, which improved PFS (HR = 0.13, 90% CI: 0.04–0.43) and OS (HR = 0.10, 90% CI: 0.02–0.36) in comparison to the chemotherapy alone. Safety was more favorable in the experimental arm [97].

The INSIGHT study was designed when the first and second-generation EGFR TKIs were available in the frontline of treatment. However, preliminary results were obtained when osimertinib became the new standard of first-line treatment care, leading to the initiation of the INSIGHT 2 phase II trial [98]. The INSIGHT 2 trial was designed to assess the efficacy and safety of the combination of tepotinib (500 mg daily) with osimertinib (80 mg daily) or tepotinib monotherapy in metastatic NSCLC patients with *EGFR* activating mutations who progressed on first-line treatment with EGFR TKIs, and who had confirmed *MET* amplification by FISH method in tissue biopsy (*MET* gene copy number of ≥5 or *MET*-to-CEP7 ratio of ≥2) or by next-generation sequencing in liquid biopsy (*MET* plasma gene copy number ≥ 2). The study enrolled 128 patients on the tepotinib plus osimertinib arm, but the primary analysis published in 2024 by Wu et al. included 98 of them. The primary endpoint showed 50% ORR (95% CI: 39.7–60.3) with a median DoR of 8.5 months, median PFS of 5.6 months, and median OS of 17.8 months. Moreover, an intracranial ORR in 24 patients with baseline brain metastases was 29.2%, of whom 25% achieved a complete response. Grade 3 or higher adverse invents included peripheral oedema in 5% of patients, prolonged QT interval in 4%, and pneumonitis in 3%. Death potentially related to the study treatment affected 3% of the studied group [99].

In addition to *MET* alterations, the C797S mutation in the *EGFR* gene is well known as the cause of secondary resistance to osimertinib, which can potentially become a new aim for targeted treatment by effective agents. In 2023, Elamin et al. shared the results of the SYMPHONY phase I/II study testing BLU-945, a next-generation oral EGFR TKI targeting T790M and C797X resistance mutations. In the trial, 108 patients received BLU-945 monotherapy, and 25 received the combination of BLU-945 with osimertinib. The authors showed a robust on-target ctDNA reduction and tumor shrinkage. The most common adverse events included nausea, headache, increased AST and ALT, as well as vomiting. The results were sufficient to plan the following studies testing BLU-945 in frontline treatment [100].

### 5.2. Treatment Independent of the Mechanism of Resistance

Due to the vast enthusiasm associated with breakthroughs provided by immunotherapy in NSCLC patients, the anti-PD-1/PD-L1 antibodies have also been tested in patients carrying activating *EGFR* mutations who progressed after EGFR TKIs, but the benefit of nivolumab nor pembrolizumab could not be demonstrated [101,102]. Contrary, in the first interim analysis of the ORIENT-31 study, sintilimab combined with chemotherapy (with or without bevacizumab) significantly prolonged PFS compared to chemotherapy alone (HR = 0.51, 95% CI: 0.39–0.67, *p* < 0.001 vs. HR = 0.72; 95% CI: 0.55–0.94, *p* = 0.016, respectively) [103].

In addition to immunotherapy, the efficiency of antibody-drug conjugates targeting different biomarkers is also not linked with the resistance mechanism to osimertinib. The TROPION-PanTumor01 phase I study was designed to assess the activity of datopotamab deruxtecan (Dato-DXD), an ADC targeting the TROP2 receptor with a payload of a topoisomerase I inhibitor in patients with solid tumors. The study included 210 NSCLC patients, 34 with actionable genetic alterations, and 29 with *EGFR-*activating mutations. 82% of these 34 NSCLC patients received three or more prior lines of treatment, and 69% of *EGFR*-mutated patients were pretreated with osimertinib. The ORR reached 35%, and the median DoR was 9.5 months. There were no new safety signals for Dato-DXD [104].

The results of TROPION-PanTumor01 justified the conduction of the TROPION-Lung05 phase II trial dedicated to NSCLC patients with actionable genetic alterations (56.9% harbored *EGFR* mutations) with progression after at least one line of targeted therapy or platinum-based chemotherapy. All patients were treated with Dato-DXD in the typical 6 mg/kg dose every 21 days. A total of 34% of patients with *EGFR* mutations reached the response with a median DoR of 7.0 months, and the safety analysis showed no new signals. The authors concluded that the obtained outcome is encouraging in this heavily pretreated population [105].

A further study evaluating ADCs was the HERTHENA-Lung01 [106] trial, a phase II study testing patritumab deruxtecan (HER3-DXD), an ADC targeting the HER3 receptor. The study enrolled 225 patients with advanced NSCLC and *EGFR* mutations who were previously treated with EGFR TKIs or platinum-based chemotherapy. In the cohort with HER3 overexpression, the administration of HER3-DXD resulted in 29.8% ORR (95% CI: 23.9–36.2), 6.4 months median DoR, 5.5 months median PFS and 11.9 months median OS. Moreover, in patients with nonirradiated brain metastases (at baseline in 30 patients), the confirmed ORR in the central nervous system was 33.3% (95% CI: 17.3–52.8) [106]. A phase III HERTHENA-Lung 02 is ongoing and this study is dedicated to patients progressing on EGFR TKIs [107].

## 6. Conclusions and Future Perspectives

To conclude, patients with *EGFR* mutations have mainly been treated with osimertinib during the last few years. The high ORR and prolonged PFS and OS justify this care standard, but most patients will eventually experience progression. Understanding the mechanism of progression is crucial as it drives important clinical decisions concerning the subsequent therapy. In the first step, patients should be evaluated after progression to identify the resistance mechanism and confirm the on-target or off-target aberrations. The methods to establish this diagnosis are not standardised, with the possibility of applying NGS and CGP rather than WES or WGS in the clinical setting. Single-gene tests are not encouraged except for some clinical trial enrollment, which is still recommended in this group of patients.

Outside clinical trials, amivantamab plus chemotherapy for patients with mutations in exon 19 or L858R substitutions in exon 21 of the *EGFR* gene is the best available option in osimertinib-resistant patients, based on MARIPOSA-2 trial results. The PALOMA-3 study showed reduced toxicity of amivantamab with subcutaneous administration, which also limits the healthcare system load. Since immunotherapy has shown inconsistent results, it is not recommended in patients with *EGFR* mutations based on available data. However, further progress is expected as many compounds are tested, with promising results for MET TKIs (savolitinib and tepotinib), next-generation EGFR TKIs (BLU-945 targeting the C797S mutation), and ADCs (patritumab deruxtecan and datopotomab deruxtecan). 

## Figures and Tables

**Table 1:** The efficacy of different treatment regimens in the MARIPOSA-2 study [85,87,88]. Abbreviation: mPFS—median of progression-free survival, ORR—overall response rate, DCR—disease control rate, mDoR—median of duration of response, IC—intracranial, mTTSP—median of time to symptomatic progression, mTTTD—median of time to treatment discontinuation, mTTST—median of time to subsequent therapy, mPFS2—median of progression-free survival on subsequent therapy, mOS—median overall survival.

	Amivanatamab + Chemotherapy	Amivanatamb + Lazertinib + Chemotherapy	Chemotherapy
mPFS	6.3 months	8.3 months	4.2 months
1-year PFS	22%	37%	13%
ORR	64%	63%	36%
DCR	87%	87%	68.4%
mDoR	6.9 months	9.4 months	5.6 months
IC mPFS	12.5 months	12.8 months	8.3 months
1-year IC PFS	50%	54%	34%
mTTSP	16.0 months	No data available	11.8 months
mTTTD	10.4 months	No data available	4.5 months
mTTST	12.2 months	No data available	6.6 months
mPFS2	16.0 months	No data available	11.6 months
mOS	17.7 months	No data available	15.3 months
18-months OS	50%	No data available	14%

## Data Availability

Not applicable.
